# Evaluation of Parents' Perceptions of the Dental and Oral Health in Children with Disability in the Bandung City

**DOI:** 10.4314/ejhs.v34i1.8

**Published:** 2024-01

**Authors:** Irsalina Nur Amalia, Eriska Riyanti, Prima Andisetyanto, Rasmi Rikmasari, Sri Tjahajawati, Yunia Dwi Rakhmatia

**Affiliations:** 1 Faculty of Dentistry, Padjadjaran University, Indonesia; 2 Department of Pediatric Dentistry, Faculty of Dentistry, Padjadjaran University, Indonesia; 3 Department of Pediatric Dentistry, Faculty of Dentistry, Padjadjaran University, Indonesia; 4 Department of Prosthodontics, Faculty of Dentistry, Padjadjaran University, Indonesia; 5 Department of Oral Biology, Faculty of Dentistry, Padjadjaran University, Indonesia; 6 Department of Prosthodontics, Faculty of Dentistry, Padjadjaran University, Indonesia

**Keywords:** Children with disability, perception, parent, children oral health

## Abstract

**Background:**

Children with disability have a risk of poor dental health because of their mental and physical limitations. They depend on caregivers in their daily life Parents have an important role in maintaining children's dental health. Parents attitudes can be influenced by parents' perceptions of children's dental health. This study explored parental perceptions regarding the dental and oral health of children with special needs in Bandung City.

**Methods:**

This study utilized a descriptive observational research using a cross-sectional survey. The subjects in this study were 239 parents who had children aged 0-18 years who were taken from 9 special schools. The variables of this study were parents' perceptions and the dental and oral health status of children with disability. Primary data were obtained through a validated questionnaire.

**Results:**

Parents' perceptions of the dental and oral health of children with disability consists of 84.94% good enough perceptions, 12.13% good perceptions, and 2.93% bad perceptions.

**Conclusion:**

Most parents have a fairly good perception of the dental and oral health of children with special needs.

## Introduction

Children with disabilities are children who experience physical, mental, social, and emotional limitations that affect their growth or development processes (1). The prevalence of children with disabilities in Bandung was 812(5.96%) (2). Children with disabilities might be at a higher risk of poor dental health owing to their mental and physical limitations (2). This is based on the findings on oral infections, periodontal disease, craniofacial birth defects, and enamel abnormalities in children with disabilities (3). However, this is not always the case in children with special needs. For example, a systematic review conducted by Moreira et al. in 2016 reported that a lower incidence of dental caries in patients with Down Syndrome was found in ten studies, and in three studies, there were no differences in dental caries experience (4). Results in Semarang City showed that 83.2% of children with disabilities experience dental caries (5). This can be caused by difficulties in maintaining dental and oral hygiene as well as the consumption of certain drugs and special diets that increase the risk of caries (4). Other factors such as the lack of dental and oral health service facilities, difficulty in routine visits to the dentist due to physical limitations, and dentists who do not want to treat children with disabilities for various reasons are obstacles for pediatric patients with disabilities that affect their oral health status (5). In addition, the social context plays a significant role in shaping an individual's oral hygiene habits and educational accomplishments. Families with low education and income pay less attention to dental care and regular preventive visits, while adolescents from higher education and greater annual income, experience an environment rich in values, social support, and resources (6,7). This environment encourages positive oral health behaviors, such as maintaining oral hygiene and brushing teeth regularly. As a result, children from higher socioeconomic status have better oral hygiene compared to children from lower socioeconomic backgrounds (7,8). Parents or caregivers often delay dental treatment because they are worried about the physical or mental condition of their children (9). According to a study conducted in Brazil, parents of children with disabilities prioritize general health over dental and oral care (10).

Children with disabilities often have difficulty learning new skills, processing complex information, or managing self-care routines to support their daily living activities, and they rely on their parents or caregivers (11). It has been reported that 62.5% of parents or caregivers admitted that their children had difficulty brushing their teeth. Therefore, parents play an important role in guiding, reminding, and providing facilities so that children can maintain their oral health and hygiene (9,12). Parents or caregivers have a direct influence on their children's oral health maintenance. However, many parents still do not pay attention to their children's dental hygiene and tend to overestimate their children's dental hygiene (13). These parents not only allow their children to brush their teeth without supervision but also trust the effectiveness of the children's tooth brushing (14). Parents' attitudes can be influenced by their perceptions of children's oral health (9,15,16). This perception is influenced by the knowledge, thoughts, attitudes, and experiences of parents or caregivers in the context of maintaining children's oral health (15).

Based on the description above, the purpose of this research was to describe parents' perceptions of the dental and oral health of children with disabilities needs in Bandung.

## Materials and Methods

This was a descriptive observational study using a cross-sectional survey. The population in this study was parents of children with disabilities in the city of Bandung, who were recorded at the health office in Bandung and listed in the data for special needs schools in Bandung. The research sample was selected using a cluster sampling technique on parents of children with disabilities in Bandung. The number of special needs schools in Bandung is 45. Six special needs schools were chosen, and 239 samples were used in this study. This sample was randomly selected from six schools, with 39-40 parents sampled from each school. The inclusion criteria in this study were parents who had children in special needs schools in Bandung city aged 0-18 years. The exclusion criteria were parents who had children born normally without any abnormalities and were unwilling to be research respondents. The variables in this study were parents' perceptions and dental and oral health status of children with disabilities.

The study utilized a questionnaire that had undergone prior validation and reliability testing, which showed that at a significance level of 5%, all statement items in the questionnaire had a validity coefficient value greater than the critical point value (0.468). This indicates that all statements in the questionnaire were valid. In addition, the reliability test on the questionnaire showed that each variable had a Cronbach's Alpha coefficient value of more than 0.700; the questionnaire used can be considered reliable.

The questionnaire used to assess parents' perceptions regarding the oral health of children with special needs was conducted using two scales: the Likert scale and the Guttman scale and consisted of four section of questions. The first section assessed the respondents identity and demographics. Section 2 consisted of nine questions to assess the condition of the child's teeth and mouth, with the first and second statements providing an overview of the general health and dental health of the children, rated on the Likert scale as either good, moderate, or bad and questions three through nine use the Guttman scale. Section 3 consisted of nine questions to assess dental care at home. Section 4 consists of 12 questions assessing the use of dental and oral health services. In sections 3 and 4, the entire assessment is conducted using the Guttman scale.

Each of these aspects is categorized into three assessment criteria: not good, fair, and good. The “not good” criterion is assigned scores between 0-33%, “fair” with scores between 34-66%, and “good” with scores between 67-100%. The results of this study provide an overview of the characteristics of parents and children, the distribution of dental and oral health frequencies in children with disabilities, criteria for assessing the condition of the teeth and mouth of children with disabilities, dental and oral care for children with disabilities, criteria for dental and oral care for children with disabilities at home, use of dental and oral health services for children with disabilities, and parents' perceptions of the dental and oral health of children with disabilities.

The results of this study are in the form of frequencies and percentages, which are presented in a tabular form. The results were analyzed using the Statistical Package for the Social Sciences (SPSS) software.

This study was conducted between April and May of 2023. This research was conducted at special needs schools in Bandung City; namely, Pancaran Iman, Az-zakiyah, Noor Rokhmah, YPLB C, Sumber Sari C, and YPDP. This study received ethical clearance from the Padjadjaran University Ethics Commission with letter number of 283/UN6.KEP/EC/2023.

## Results

This study comprised 239 parents residing in Bandung, each having a child with disabilities. The characteristics of parents and children with disabilities are shown in Table 1. Among the disability categories, intellectual disorders were predominant and the majority of the children belonged to age 13-18 years. Additionally, male children constituted the larger proportion (60.67%).

**Table 1:** Characteristics of parents and children with disabilities

Variable		Frequency	Percent
Abnormalities	Intellectual Disorders	164	68.62
	Physical Disorders	8	3.35
	Sensory Disorders	9	3.77
	Mental disorders	54	22.59
	Combination	4	1.67
Child Age	0-6 years old	3	1.26
	7-12 years old	107	44.77
	13-18 years old	129	53.97
Gender	Woman	94	39.33
	Man	145	60.67
Parents Age	28-34 years old	10	4.18
	35-41 years old	64	26.78
	42-48 years old	77	32.22
	49-55 years old	69	28.87
	56-62 years old	13	5.44
	63-69 years old	6	2.51
Parents' Last Education	Elementary school	32	13.39
	Middle school/equivalent	33	13.81
	High school/equivalent	102	42.68
	Diploma	16	6.69
	Undergraduate and level at on it	56	23.43
Parents' job	Not Yet/Not Working	1	0.42
	Housewife	77	32.22
	Civil servant	13	5.44
	TNI/Polri	3	1.26
	Retired	3	1.26
	Private sector employee	50	20.92
	BUMN/BUMD employees	2	0.84
	Medical personnel	1	0.42
	Self-employed	34	14.23
	Teacher	5	2.09
	Other	50	20.92

The average age of parents of children with disabilities was most commonly found in the age group of 42-48 years (32.22%). The majority of parents had high school education/equivalent (42.68%), and most parents worked as housekeepers (32.22%).

Parents' perceptions of dental and oral health in children with disabilities was assessed based on three aspects, which were the condition of the child's teeth and mouth, dental and oral care at home, and the use of dental and oral health services. An overview of the characteristics of dental and oral health in children with disabilities is shown in Table 2.

**Table 2:** Frequency Distribution and Criteria for Assessing the Dental and Oral Conditions in Children with Disabilities in the City of Bandung

Frequency Distribution			

Statement	Good	Moderate	Bad
General health	165	72	2
Dental and oral health	51	178	10

**Frequency Distribution**			

**Statement**		**Yes**	**No**

Never had a toothache		94	145
No cavities		69	170
Have cavities less than or equal to 2 teeth		110	129
No bad breath		103	136
No gingival disease		113	126
No crowded teeth		120	119
No grinding habits		131	108

Criteria		

Oral and Mouth Condition	Frequency	Percent
Not good	7	2.93	
Fair	145	60.67	
Good	87	36.40	
Total	239	100	

Table 2 shows that most parents (69.04%) assessed their children's general health as good. However, in dental and oral health, the majority of parents considered their child's dental and oral health to be moderate (74.48%). In addition, 170(71.13%) children with disabilities experienced dental cavities issues, and about 129(53.97%) children have two or fewer cavities.

The majority of parents assess that their children have fair teeth and mouth conditions (60.67%). However, there were still 7 children with disabilities (2.93%) whose teeth and mouth were not in good condition. An overview of dental and oral care at home for children with disabilities presented in Table 3.

**Table 3:** Frequency distribution and Criteria for dental and oral care for children with disabilities at home

Frequency Distribution		

Question	Yes	No
Can brush their own teeth	152	87
Parents helping children brush their teeth	119	120
Parents should remind children to brush their teeth	204	35
Brushing teeth regularly	195	44
Brushing teeth twice a day	130	109
Brushing teeth after breakfast	106	133
Brushing teeth before bed	149	90
Using mouthwash	19	220
Does not like sweet food	89	150

Criteria		

Oral Care at Home	Frequency	Percentage (%)
Not good	24	10.04
Fair	127	53.14
Good	88	36.82
Total	239	100

Table 3 shows that most children with disabilities were able to brush their teeth. Half of the studied children with disabilities were not assisted by their parents when brushing their teeth. Thus, 204(85.36%) children with disabilities still needed to be reminded to brush their teeth. Most children with disabilities brushed their teeth regularly. However, only 130 (54.39%) children with disabilities brushed their teeth twice daily.

Only a few children used mouthwashes (7.95%), and the remaining 220 children (92.05%) did not use mouthwashes. The majority of children with disabilities (62.76%) liked eating sweet foods such as candy and chocolate. Most children with disabilities in Bandung received good dental and oral care at home. An overview of the use of dental and oral health services in children with disabilities is presented in Table 4.

**Table 4:** Frequency distribution and criteria of the use of dental and oral health services for children with special needs

Frequency Distribution		
	Yes	No
Questions		
Been to the dentist.	141	68
Go to the dentist at the hospital.	98	141
Go to the dentist in private practice.	60	179
Last time go to the dentist for routine dental checks.	32	207
Last time go to the dentist to clean teeth.	36	203
Last time go to the dentist to fill the teeth.	34	205
Last time go to the dentist because of problems with gums.	37	202
Last time go to the dentist to extract a tooth.	67	172
Last time go to the dentist because of a toothache.	83	156
Last time go to the dentist for reasons other than those mentioned above	44	195
For the last 12 months my child has been to the dentist.	66	173
Visit the dentist regularly.	9	230
Have dental and oral health insurance.	38	201
Never missed school because of problems with his teeth and mouth.	111	128
Not afraid to see the dentist.	116	123
Not going to the dentist because access to the dentist's office is difficult (far/difficult to transport/bad roads).	10	229
Not going to the dentist because it's expensive.	82	157
Not going to the dentist because of lack of information (don't know where the dentist practices/don't know when to go to the dentist).	36	203
Not going to the dentist because the dentist's service is unsatisfactory.	8	231

Criteria		

The Use of Dental and Oral Health Services	Frequency	Percent
Not good	102	42.68
Fair	121	50.63
Good	16	6.69
Total	239	100

The table shows that more than 50% of children with disabilities visited the dentist. The majority of children with disabilities needed to visit a dentist at the hospital (41%). Only a few parents took their children to the dentist for routine checks. Only 66 (27.62%) parents had taken their children to the dentist in the last 12 months. The low intensity of parents taking their children to the dentist can also be seen from the number of children who had dental and oral health insurance, only 38 children (15.90%). More than 50% of children with disabilities in Bandung missed school because of problems with their teeth and mouth.

The reasons why parents do not bring their children to the dentist can be seen in several aspects, including access, costs, information, and services from dentists. The most common reason for parents of children with disabilities to not bring their children to the dentist was that their children were afraid of seeing a dentist (51.46%). Then, 82 parents (34.31%) felt that going to the dentist was expensive, while the remaining 157 parents (65.59%) did not consider visiting the dentist to be expensive.

Parents believed that access to information related to dentists in Bandung is not difficult to obtain. In the follwoing table, we also can find that the majority of children with disabilities in the city of Bandung used dental and oral health services quite well, as many as 121 children (50.63%).

Table 5 shows the parents' perceptions of the dental and oral health of children with disabilities. Parents had a fairly good perception of the dental and oral health of children with disabilities as much as 84.94% while 12.13% had a good perception and the remaining 2.93% had a poor perception of the dental and oral health of children with disabilities in the city of Bandung.

**Table 5:** Criteria for assessing parents' perceptions of the dental and oral health of children with disabilities in the City of Bandung

Parents Perception	Frequency	Percent
Not good	7	2.93
Fair	203	84.94
Good	29	12.13
Total	239	100

## Discussion

Maintaining optimal dental and oral health in children, particularly those with disabilities, is crucial for their overall growth and development (17). According to James A. Wedell, et al. (18), it is essential for parents to oversee the dental hygiene routine of children with disabilities (18). Within this study, 85.36% of parents always reminded their children to brush their teeth. Surprisingly, 50.21% of parents reported not directly assisting their children during brushing sessions, potentially impacting their children's oral hygiene due to physical or mental limitations (17).

Interestingly, 81.59% of children with disabilities brushed their teeth regularly, and the criteria for dental care at home were quite good, as much as 53.14%. On initial assessment, these factors appear conducive to favorable dental and oral health outcomes. However, in this study, 71.13% of the children had cavities, 52.72% had gum problems, and 56.90% had bad breath. This phenomenon can be caused by improper dental and oral care at home, such as inappropriate tooth brushing habits, such as improper tooth brushing techniques, the use of unsuitable brushing tools, and irregular brushing frequency. (19,20).

In this study, almost half of the children did not brush their teeth twice a day (45.61%) and 55.65% did not brush their teeth after breakfast. Limanto et al.'s research (19) revealed that 61.1% of fifth and sixth-grade elementary school students, practicing inadequate brushing (<2 times/day) and improper techniques, experienced dental caries (19). Brushing teeth is an attempt to clean food residues in the teeth and mouth (19). According to Imran and Nakurniawati, the ideal toothbrush frequency is 2–3 times per day (after breakfast, after lunch, and before going to bed) (20). Incorrect brushing techniques can result in food residue accumulation or injuries to the gums and oral mucosa (20). The proper tooth-brushing method involves brushing all tooth surfaces, interdental spaces, and the tongue to eliminate food debris and deter bacterial colonization or biofilm formation on the teeth (20).

Another contributing factor to dental and oral issues is the consumption of sugary foods (21). In this study, children with disabilities displayed a strong preference for sweet foods. Research conducted by Rekawati and Frisca (21) similarly demonstrated that 74.6% of elementary school children aged 6–11 years, who frequently consumed sweet foods, suffered from dental caries (21). hese cariogenic foods, characterized by their sweetness, softness, and stickiness, adhere to tooth surfaces, making them challenging to clean (21). Consequently, oral flora fermentation occurs, leading to increased mouth acidity, enamel damage, and subsequent dental caries (21). Furthermore, food residue accumulation on teeth can foster biofilm formation, contributing to halitosis (21). Dietary analysis is a caries prevention measure that aims to avoid cariogenic diets and is one of the main programs of preventive dentistry for children with disabilities (22). Diet and nutritional intake play a significant role in caries occurrence by influencing the microbial composition and virulence factors within dental plaque, as well as affecting tooth defense mechanisms, supportive oral tissues, and saliva components (22).

Bad oral habits such as grinding teeth or bruxism can also cause dental caries (23). Within this study, half of the children with disabilities exhibited a tendency to grind their teeth. Research by Regiawan et al. (24) showed that 64% of respondents who had bruxism habits had dental caries and malocclusion in the mouth (24). Bruxism results in shortened tooth crowns and thinning of tooth enamel due to the grinding action, which can lead to caries and heightened tooth sensitivity (24). Moreover, bruxism has the potential to cause temporomandibular joint (TMJ) disturbances, consequently leading to malocclusion (24).

Children with disabilities need assistance from the people around them, especially their parents in maintaining dental and oral health (18). In this study, the majority of parents rated their children's dental and oral health as moderate. In addition, the results of the objective assessment of dental and oral health criteria in this study were in the majority category which is quite good. Despite parents appraising their child's oral and dental condition as quite good, there are still children who experience dental and oral problems such as cavities, gum problems, bad breath, and toothaches. This can reflect that parents feel that dental and oral problems in their children are normal and problems that occur in the teeth and mouth are considered harmless.

Parents' perceptions greatly influence dental and oral health outcomes in children, especially in children with disabilities (25). In this study, only 12.3% of parents' perceptions of dental and oral health were good. According to Pratamawari and Hadid (26), the better a person's perception of dental and oral health, the more often a person visits the dentist to reduce the incidence of dental and oral diseases (26). Parents' perceptions of the dental and oral health of children with disabilities can be influenced by their level of education, knowledge, and attitudes in making decisions regarding the interests of dental and oral health in children with disabilities (26). According to Afiati et al.(27) people with higher levels of education have good knowledge and behavior about health so they can improve healthy living behaviors (27).

An interesting phenomenon in this study was that the majority of parents' last education level was high school/equivalent. This reflects that some of the parents in this study had a fairly high level of education, but there were still many cases of dental and oral problems in the children in this study. Education level does not fully guarantee knowledge of dental and oral health (28). This means that a person needs to obtain information about dental and oral health knowledge from the correct sources so that parents are not mistaken in determining their attitude (28).

Information regarding proper dental and oral health practices can be acquired from various healthcare facilities such as health centers, hospitals, and dental practices (28,29). In this study, almost half of the utilization of dental and oral health services for children with disabilities fell into the unfavorable category. According to James Wedell et al, (18) parents must follow up to improve the dental and oral health conditions of children with disabilities by visiting the dentist (18). However, in this study only 15.06% of parents had routine dental and oral care at the dentist, 86.61% had never taken their children to the dentist in the last 12 months, and 41% had never taken their children to the dentist. The reason is that half of children with disabilities were afraid of going to the dentist, and 34.31% parents felt that going to the dentist is expensive. This can affect parental knowledge about dental and oral health in children with disabilities and has significant implications for parents' motivation to maintain healthy teeth and mouth. This study aligns with the research of Maharani and Rahardjo (30) which stated that only 0.74% of respondents used dental and oral health services (30). The lack of intensity of parents taking their children to the dentist can also be seen in the number of children who have dental and oral health insurance which is only 15.90%. This indicates that urgent support from dental and oral health insurance providers is needed to ensure dental and oral health services for children with disabilities. However, until now private health insurance providers and state health insurance have not provided special services for guaranteeing dental and oral health for children with disabilities.

The limitations of this study include not assessing the economic factors of the respondents, therefore the research results are less specific because the relationship between perceptions and economic factors cannot be seen. This study was also not accompanied by direct clinical data, and the limitation of this research is its inability to evaluate the oral health status of children aged 0-5 years, since children in this age range are not yet registered in special needs schools. Furthermore, the use of questionnaires as a research instrument also makes it possible for recall bias, as participants may not always provide accurate or truthful responses. These limitations can be addresses in future research.

Suggestions regarding the results of this study to improve dental and oral health in children with disabilities also need support from parents and dentists (31). Parental support is expected to have good perceptions and motivation about the dental and oral health of children with disabilities to provide a positive attitude, such as correct dental health care at home, obtaining appropriate and reliable information, and utilizing dental and oral services at health facilities, in hospitals, health centers and dentist. Parents of children with disabilities who have difficulty in maintaining the health of their children's teeth can assist in the care of their children's teeth. In addition, dentists or health service providers need to provide counseling to parents and teachers regarding oral and dental health in schools. Dentists also need to provide full support to improve the dental and oral health status of children with disabilities through comprehensive and holistic services.

Based on the results of research on parents' perceptions of children with disabilities in Bandung, it can be concluded that most parents of children with disabilities within the age range of 6-18 years tend to have fairly good perceptions of the dental and oral health of children with disabilities.
